# Putative pleiotropic effects of the knockdown resistance (L1014F) allele on the life-history traits of *Anopheles gambiae*

**DOI:** 10.1186/s12936-021-04005-5

**Published:** 2021-12-20

**Authors:** Adandé A. Medjigbodo, Luc S. Djogbénou, Oswald Y. Djihinto, Romaric B. Akoton, Emmanuella Abbey, Rosaria M. Kakossou, Eric G. Sonounameto, Esther B. J. Salavi, Laurette Djossou, Athanase Badolo

**Affiliations:** 1 Tropical Infectious Diseases Research Centre (TIDRC), University of Abomey-Calavi, 01BP 526 Cotonou, Benin; 2 Laboratory of Fundamental and Applied Entomology, University Joseph Ki-Zerbo, BP 7021 Ouagadougou 03, Burkina Faso; 3 Regional Institute of Public Health/University of Abomey-Calavi, BP 384 Ouidah, Benin; 4 Department of Vector Biology, Liverpool School of Tropical Medicine, Pembroke Place, Liverpool, L3 5QA UK

**Keywords:** *Kdr*^R^ allele, Fitness effects, Life-history traits, *Anopheles gambiae*, Malaria

## Abstract

**Background:**

Existing mechanisms of insecticide resistance are known to help the survival of mosquitoes following contact with chemical compounds, even though they could negatively affect the life-history traits of resistant malaria vectors. In West Africa, the knockdown resistance mechanism *kdr*^*R*^ (L1014F) is the most common. However, little knowledge is available on its effects on mosquito life-history traits. The fitness effects associated with this knockdown resistance allele in *Anopheles gambiae *sensu stricto (*s.s.*) were investigated in an insecticide-free laboratory environment.

**Methods:**

The life-history traits of Kisumu (susceptible) and KisKdr (*kdr* resistant) strains of *An*. *gambiae s.s.* were compared. Larval survivorship and pupation rate were assessed as well as fecundity and fertility of adult females. Female mosquitoes of both strains were directly blood fed through artificial membrane assays and then the blood-feeding success, blood volume and adult survivorship post-blood meal were assessed.

**Results:**

The *An. gambiae* mosquitoes carrying the *kdr*^R^ allele (KisKdr) laid a reduced number of eggs. The mean number of larvae in the susceptible strain Kisumu was three-fold overall higher than that seen in the KisKdr strain with a significant difference in hatching rates (81.89% in Kisumu *vs* 72.89% in KisKdr). The KisKdr larvae had a significant higher survivorship than that of Kisumu. The blood-feeding success was significantly higher in the resistant mosquitoes (84%) compared to the susceptible ones (34.75%). However, the mean blood volume was 1.36 µL/mg, 1.45 µL/mg and 1.68 µL/mg in Kisumu, homozygote and heterozygote KisKdr mosquitoes, respectively. After blood-feeding, the heterozygote KisKdr mosquitoes displayed highest survivorship when compared to that of Kisumu.

**Conclusions:**

The presence of the knockdown resistance allele appears to impact the life-history traits, such as fecundity, fertility, larval survivorship, and blood-feeding behaviour in *An. gambiae*. These data could help to guide the implementation of more reliable strategies for the control of malaria vectors.

## Background

Malaria is still one of the most devastating parasitic diseases, especially in tropical regions worldwide. This disease spreads in 96 countries in America, many parts of Asia and most of Africa [1]. Malaria is transmitted through the bite of infected female *Anopheles* mosquitoes, which carry the infection by protozoan parasites *Plasmodium* species to humans [2]. In most regions in sub-Saharan Africa, mosquito species including *Anopheles gambiae *sensu stricto (*s.s.*)*, Anopheles arabiensis*, *Anopheles coluzzii*, and *Anopheles funestus* are the main vectors that transmit malaria parasites [3–5]. Since an effective malaria vaccine is yet to become available, vector control remains the main strategy for the prevention of malaria transmission [6]. Indeed, long-lasting insecticide-treated nets (LLINs) and indoor residual spraying (IRS) remain the backbone of malaria vector control and have been shown to contribute to malaria control through the reduction of human-vector contact [7]. Unfortunately, insecticide resistance to pyrethroids (permethrin, deltamethrin) and other classes of insecticides has been reported in *An. gambiae,* the main malaria vector in several African countries [8–14]*.* The major insecticide resistance mechanisms in *An. gambiae* consist of target sites insensitivity (*ace-1*^R^ and *kdr*^R^) and increased metabolic activity of detoxifying enzymes [15–20]. In *An. gambiae s.s.*, mutations related to pyrethroids and dichlorodiphenyltrichloroethane (DDT) resistance are located mainly at codon 1014 within the transmembrane segment 6 of domain II in the *Voltage-gated sodium channel* (*Vgsc*) gene. These mutations lead to a change of leucine to either phenylalanine (L1014F) or serine (L1014S) [21, 22]. Further, additional mutation at position 1575 of the linker between domains III-IV in the *Vgsc* resulting in asparagine-to-tyrosine substitution (N1575Y) has been found occurring solely on a L1014F-bearing haplotype [23]. Recent studies carried out in Benin [24], Ivory Coast [25] and Burkina Faso [26] have shown that the L1014F allele frequency is almost fixed in wild *An. gambiae* mosquitoes. However, little is known about the fitness cost induced by this homozygous resistance allele in the malaria vector *An. gambiae*.

Although resistance alleles confer the potential of surviving particular insecticide exposures to mosquitoes, it is often assumed that they may also influence various fitness-related traits of mosquitoes (e.g., trophic behaviour, fecundity, fertility, parasite transmission, longevity, and larval survivorship) in the presence or absence of insecticide selection pressure [27]. Therefore, better understanding the effects of resistance alleles on the most important life-history traits of mosquitoes appears crucial to improve malaria vector control interventions.

Several studies have shown that insecticide resistance mechanisms can confer detrimental effects on reproductive fitness, host-seeking, feeding and mating behaviours in *Anopheles* mosquitoes [28–30] as well as in some *Aedes* [31–33] and *Culex* mosquitoes [34–36]. Decreased longevity and increased larval survivorship have also been observed in insecticide-resistant strains of *Aedes aegypti*, *Culex pipiens* and *An. gambiae* [31, 37–40]. A study carried out by Platt et al*.* [30] revealed that *kdr*^R^ heterozygous males *An. coluzzii* were more likely to successfully mate than homozygote-resistant ones, illustrating a deleterious effect of homozygote-resistant *kdr*^R^ allele on *An. coluzzii* paternity success. Also, they were more competitive compared to homozygous-susceptible mosquitoes indicating a heterozygous fitness advantage [30]. Furthermore, it was demonstrated that pupae of *An. gambiae* homozygous for *ace-1*^R^ (G119S) allele were more likely to die during the pupation stage than those of the susceptible strain [40]. All these studies highlight the variability of mosquito life-history traits according to species and the effects of specific insecticide resistance mechanisms on these traits.

Herein, the relative effects of *kdr*^R^ (L1014F) allele on reproductive success, larval survivorship, blood-feeding behaviour, and adult survivorship post-blood meal in *An. gambiae s.s.* were evaluated.

## Methods

### Mosquito strains and rearing

Two laboratory reference strains of *An. gambiae s.s.* were used. The insecticide-susceptible reference strain Kisumu, sampled from Kenya the early 1950s and was maintained at insectary [41]. The KisKdr strain, which is homozygous [*kdr*^RR^] for the L1014F allele and resistant to both pyrethroids and organochlorines, was obtained by introgression of the *kdr*^R^ (L1014F) allele into the Kisumu genome [42]. This strain has the same genetic background as Kisumu [*kdr*^SS^] and was free of metabolic resistance.

In order to investigate the role of *kdr*^R^ (L1014F) allele in *An. gambiae s.s.* blood-feeding behaviour, heterozygote [*kdr*^RS^]-resistant mosquitoes were obtained by crossing Kisumu females [*kdr*^SS^] with KisKdr males [*kdr*^RR^] and Kisumu males with KisKdr females encoded F1-1 (♀Kisumu X ♂KisKdr) and F1-2 (♂Kisumu X ♀KisKdr), respectively. For routine rearing in the insectary at the Regional Institute of Public Health/ University of Abomey-Calavi (Benin), these strains were reared under soft conditions (insecticide-free laboratory environment) in a climate-controlled room at a temperature fixed at 27 °C (± 0.2), a relative humidity of 70% (± 8) and 12:12 light and dark period. Larvae were reared in plastic trays (about 30 × 20 cm) and fed with TetraMin Baby fish food. Pupae were collected and placed in small plastic cups inside a fresh cage for adult emergence. Adult mosquitoes were kept in 30 × 30x30 cm insect cages (produced locally) and continuously supplied. Mosquitoes were fed ad libitum on 10% honey solution (made with deionized water) until they were ready to be used for further assays. Female individuals were blood-fed on laboratory rabbits (used for the purpose of blood-feeding mosquitoes) twice a week. Gravid females were allowed to oviposit in plastic petri dishes containing a water-soaked cotton covered with filter paper. The eggs were collected and put in plastic trays containing dechlorinated water (1 L per tray) for hatching.

### Female reproductive success assessment

Three days after emergence from the larval-rearing conditions described, 180 *An. gambiae* females of both KisKdr (n = 90) and Kisumu (n = 90) strains were blood-fed on a laboratory rabbit. The gravid mosquitoes of each strain were individually transferred into plastic cups containing wet Whatman filter paper for oviposition. They were allowed to feed on 10% honey solution until egg laying. The number of females that laid eggs was recorded and the eggs were counted under a stereomicroscope (Leica Microsystems EZ4HD). Egg batches (from individual females) were transferred in separate plastic trays (about 10 cm diameter) filled with dechlorinated water and the number of hatched larvae was recorded. The experiments were performed two times.

### Larval survival assessment

The larvae from each mosquito strain reared in insecticide-free laboratory conditions as described, were used for the survival assays. To assess larval mortality associated with *kdr*^R^ (L1014F) allele in each mosquito strain, assays were performed as described by Yahouédo et al. [43]. In total, 480 first instar larvae (L1) of each mosquito strain were used. For each replicate, 32 larvae were pipetted into a 50 mL graduated plastic beaker (9 cm diameter). The beaker was filled with dechlorinated water to the 32 mL mark and larvae were then poured into a new petri dish. The petri dishes remained covered with the lids and their positions were changed every day to compensate for any localized differences that may exist on the rack. Petri dishes were used in order to reduce variation in larval growth rate. Every day, the larvae of each petri dish were fed with 640 µg of TetraMin Baby fish food. Water was changed every two days to reduce the effect of pollution. The petri dishes containing larvae were inspected once daily and the dead pupae or larvae were recorded and removed. Daily mortality of larvae was monitored until the last one reached pupal stage. The experiments were performed three times.

### Assessment of blood-feeding behaviour

Membrane feeding assays (MFAs) previously described by Kristan et al. [44] were performed to blood-feed the mosquitoes. The 3–5-days old females of Kisumu (n = 495), KisKdr (n = 200) and those from the crossings, namely F1-1 (n = 95) and F1-2 (n = 105), were used in three different experiments. Mosquitoes were glucose-starved (with access to water-soaked cotton) for 24 h and the batches of 25 individuals were separately exposed for 30 min to membrane feeders containing the blood sample pre-heated following procedures described in [45]. The fully blood-fed mosquitoes were scored 24 h later and were kept for survivorship assessment post-blood feeding.

A portion of the blood-fed mosquitoes was used to assess the blood meal size using a spectrophotometer (MULTISCAN GO, Thermo Scientific) as previously described [46]. Each experiment using at least 30 individuals per strain, was performed three times.

### Mosquito longevity post-blood meal

After the blood-feeding assays, successfully blood-fed females from Kisumu (n = 172), KisKdr (n = 168), F1-1 (n = 71) and F1-2 (n = 90) were transferred into brand-new disposable paper cups (an average 10 females per cup) and were allowed to feed on 10% honey solution. The mortality was recorded daily until the death of the last mosquito.

### Data analysis

Data were recorded in appropriate designed forms, entered into Microsoft Excel for data cleaning and exported to R statistical software version 3.4.4 [47] and GraphPad Prism 8.0.2 software (San Diego, CA, USA) for analysis. The normality of data distribution was checked using Shapiro Wilk test [48].

Fecundity of each mosquito strain was assessed as the total number of eggs over the total number of females that contributed to oviposition. A correlation between *kdr*^R^ genotype and fecundity was calculated using negative binomial model (NBM) defined as follow: log (Ov) = Genotype + *ε* where Ov is the number of eggs/female; Genotype is the two-level factor corresponding to the different genotypes tested; *ε* is the error parameter which follows a negative binomial distribution. For each mosquito strain, fertility was evaluated as percentage of hatched larvae by dividing the total number of first instar larvae over the total number of eggs. A correlation between *kdr*^R^ genotype and fertility was calculated using NBM, defined as follow: log (Ha) = Genotype + *ε* where Ha is the percentage of larvae/egg batch. Descriptive statistics were used to calculate pupation percentage (number of pupae/number of first instar larvae), blood-fed mosquito percentage (number of blood-fed mosquitoes/number of exposed mosquitoes). The Chi-square independence test was performed to compare proportions using the R statistical software [47]. The Mann–Whitney procedure was used to compare the means between mosquito strains. For the larval and blood-fed females survivorships, differences in the computed survival curves of Kisumu and KisKdr strains were analysed using Kaplan–Meier pair-wise comparisons [49]. The Log-rank test was performed to evaluate the difference in survival time between the mosquito strains [50]. Differences in larval survival time and in adult survival time post-blood meal between the two genotypes were tested using Cox proportional hazards regression model (Cox model) with a binomial error distribution. The models were calculated as follows: Survival = Genotype + *ε*, where Survival is a proportion of dead larvae or adults; Genotype is the two-level factor corresponding to the different genotypes tested; *ε* is the error parameter which follows a binomial distribution. The pupae were censored in the larval survivorship analysis. The significance of differences in blood-feeding rates between the genotypes was assessed with the following generalized linear models (GLM): Fed = Genotype + *ε*, where Fed is the blood-fed status; Genotype is a three-level factor corresponding to the different genotypes tested ([*kdr*^SS^], [*kdr*^RS^] and [*kdr*^RR^]); *ε* is the error parameter which follows a binomial distribution. All these analyses were set at significance threshold of *p* < 0.05.

## Results

### Reproductive success

**Fig. 1:**
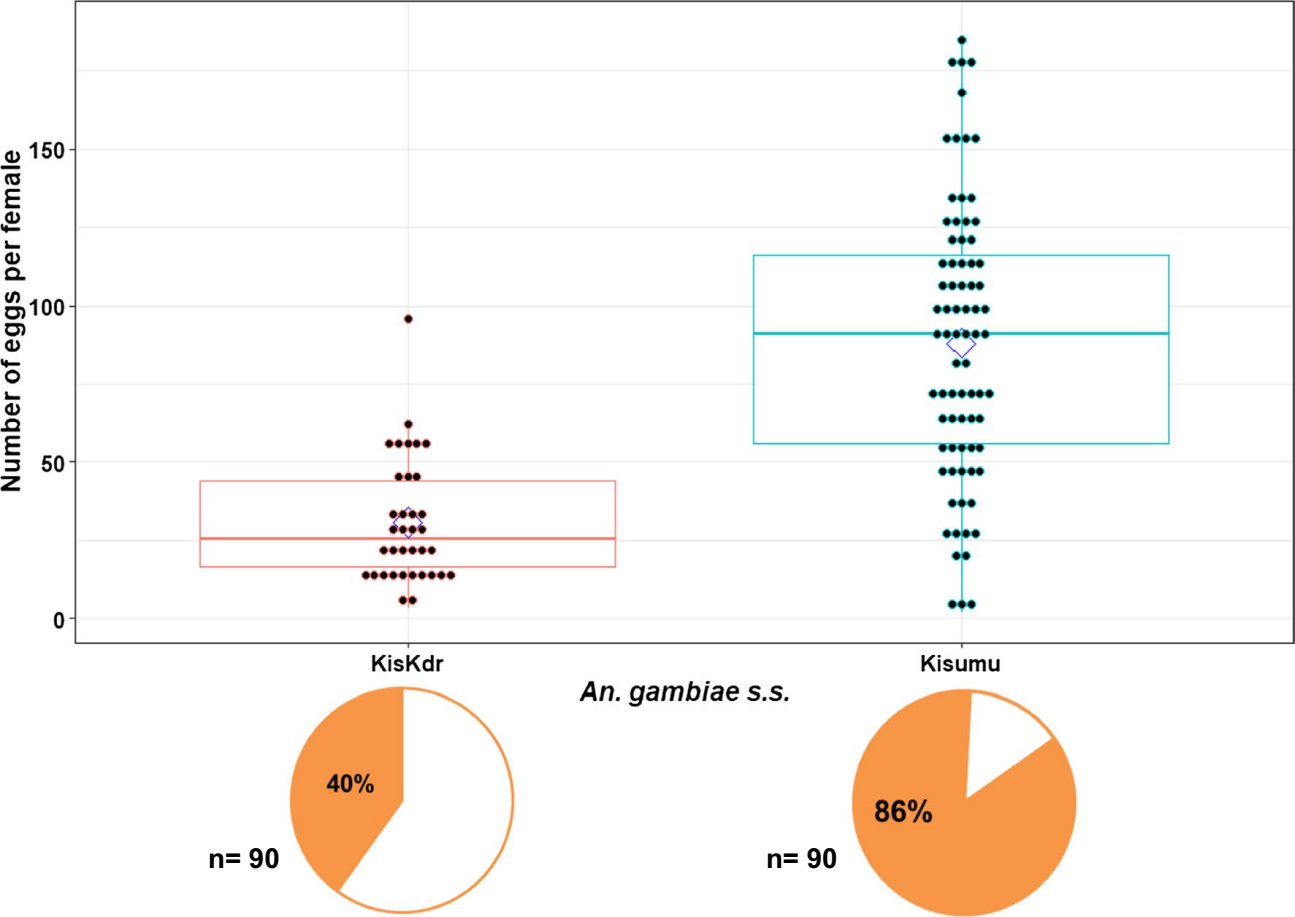
Fecundity in KisKdr and Kisumu strains. Each dot denotes the number of eggs laid by each female in each strain. Only females that laid at least one egg were included. A diamond point represents the mean number of eggs in each strain and the box plots represent the median and its 25 and 75% interquartile. Pie charts represent percentages of mosquito females that laid eggs in each strain. (n) indicates the total number of mosquito females subjected to the oviposition. Significant difference was observed in fecundity between both Kisumu and KisKdr mosquito females (*p*= 1.07x10^−10^)

**Fig. 2:**
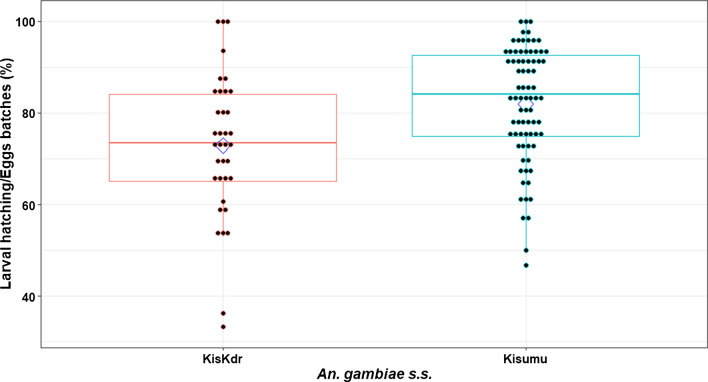
Kisumu and KisKdr larval-hatching percentages. Each dot denotes the percentage of larvae hatched from individual female eggs batch within each strain. A diamond point represents the mean percentage of larvae hatched from each strain and the box plot represents the median and 25% and 75% interquartile ranges. Significant difference was observed in hatching rates between both Kisumu and KisKdr females (*p* = 0.02 × 10^–1^)

The mean number of eggs laid per mosquito female (fecundity) and the average larval hatching rate (fertility) were significantly different between the two strains (30.72 ± 19.92 eggs/KisKdr female *vs* 87.98 ± 44.51 eggs/Kisumu female, *p* = 1.07 × 10^–10^; Fig. 1) and (72.89 ± 15.7% hatched larvae/KisKdr female *vs* 81.89 ± 12.4% for Kisumu female, *p* = 0.02 × 10^–1^; Fig. 2). Moreover, the KisKdr female fecundity and fertility decreased by 1.05 (GLM.NB: *F* = 58.21, *Δdf* = 1, *p* = 8.71 × 10^–12^) and 0.12 (GLM.NB: *χ*^*2*^ = 1062, *Δdf* = 1, *p* = 0.01 × 10^–1^), respectively, when compared to those of Kisumu females. Overall, the reproductive success of KisKdr [*kdr*^RR^] females was significantly lower than that of Kisumu [*kdr*^SS^] females.

### Larval survivorship

**Fig. 3:**
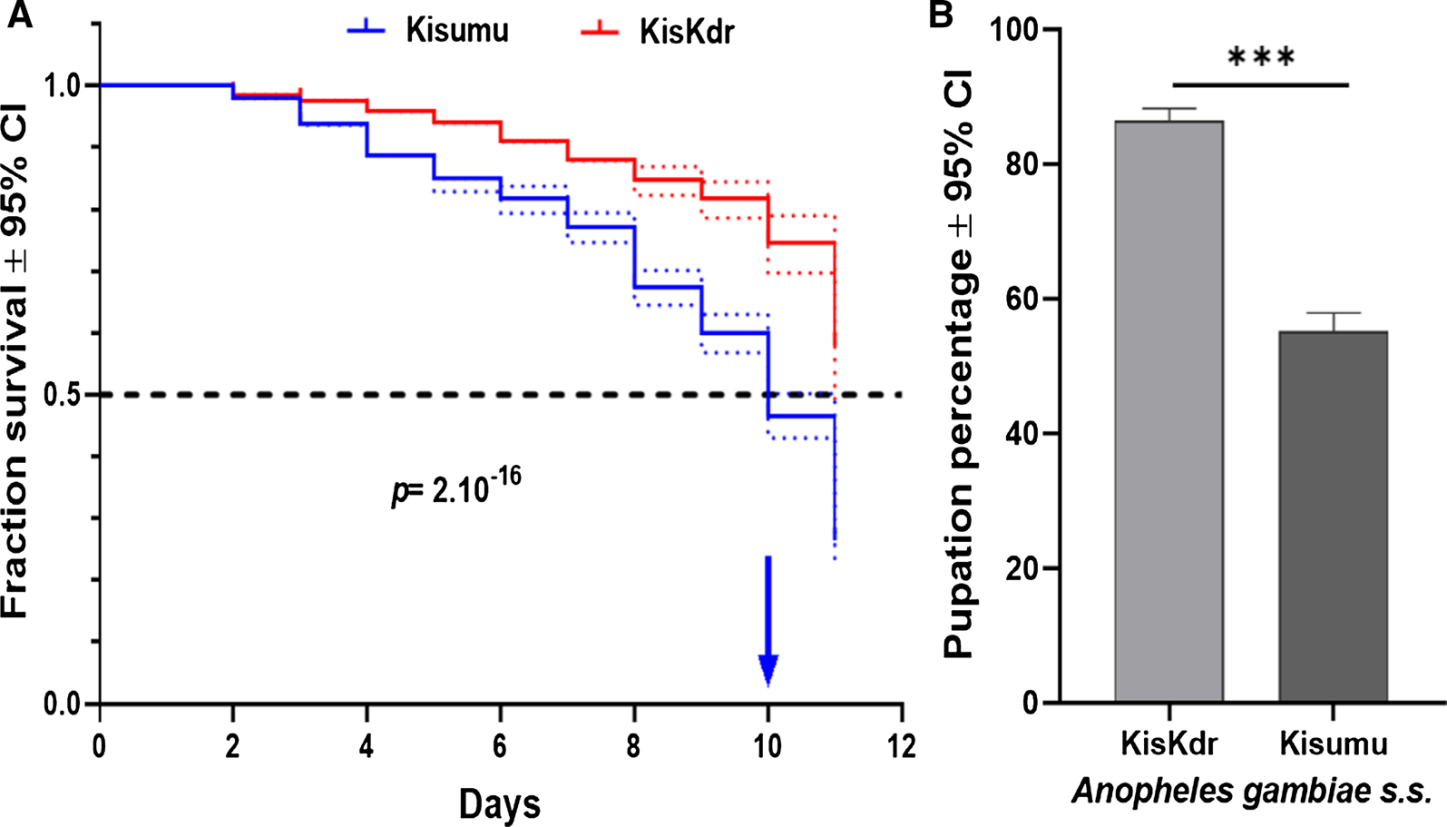
KisKdr and Kisumu larvae longevity **A** and pupation percentages **B**. **A** Dotted lines are 95% confidence intervals (CIs) around the respective survival curve. Arrow indicates the median survival time. **B** Error bars are 95% confidence intervals (CIs) around each percentage. ***indicate *p* = 0.01 × 10^–2^

The median survival times of Kisumu and KisKdr larvae were, respectively, 10 days and 11 days (Fig. 3A). However, the survival time of Kisumu larvae was significantly shorter than that of KisKdr larvae (Log-rank test: *χ*^*2*^ = 110, *Δdf* = 1, *p* = 2.10^–16^). Furthermore, more than 50% of KisKdr larvae were still alive and have reached the pupal stage at the end of the larval following-up period (Fig. 3A). The risk of death of individual larvae when bearing *kdr*^R^ allele at homozygote state [*kdr*^*RR*^] is reduced by a factor of 59% compared to homozygote susceptible larvae [*kdr*^SS^] (Cox model: likelihood ratio test (LRT): *χ*^*2*^ = 114.7, *Δdf* = 1, *p* = 2.10^–16^). Consequently, pupation rate in KisKdr females was significantly higher (85.84%, CI_95%_ = [84.12–87.75]) than that recorded for Kisumu strain (54.05%, CI_95%_ = [51.34–56.74]) (Fig. 3B).

### Blood-feeding success

**Fig. 4:**
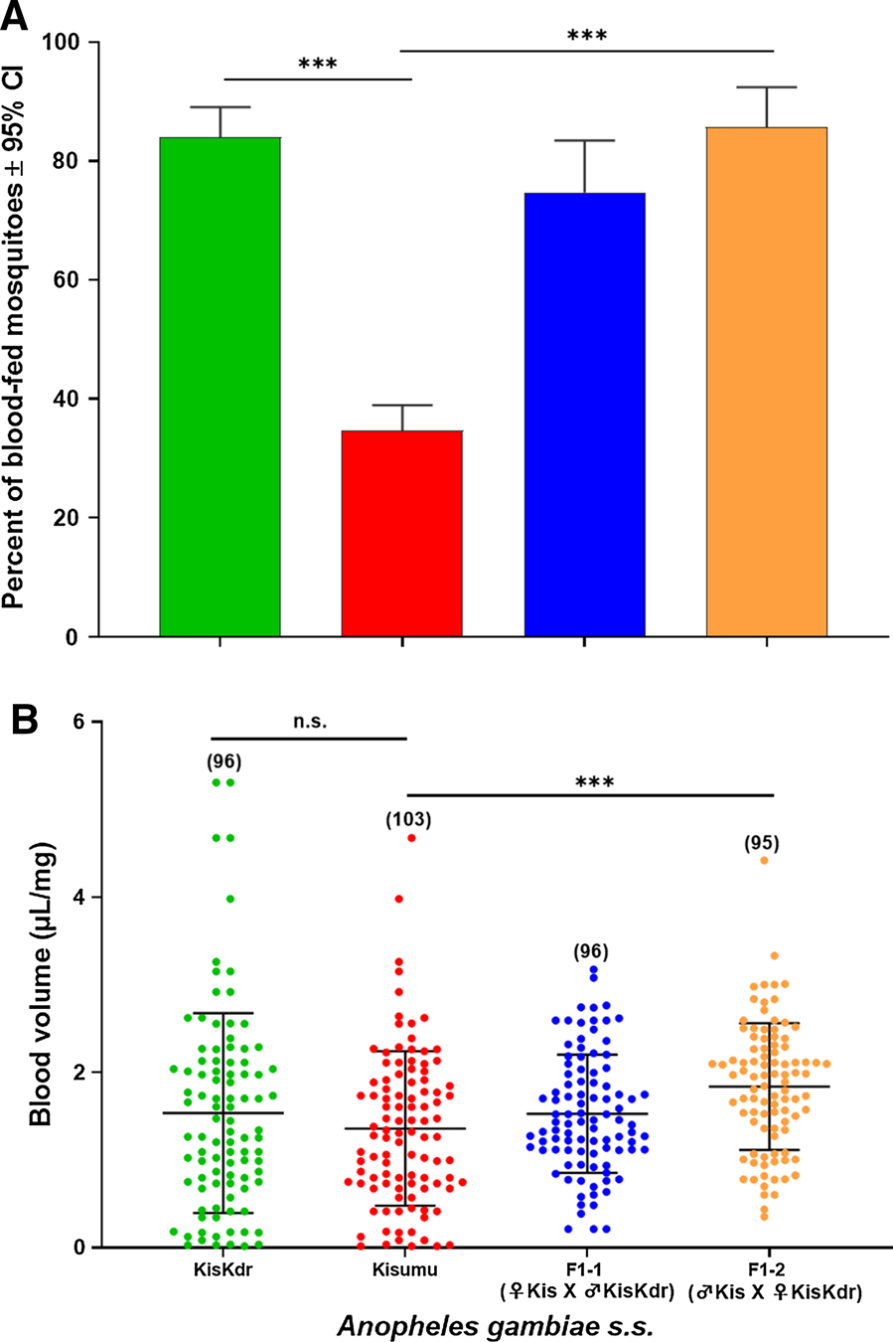
Percentages of blood-fed females **A** and blood meal size **B** in *kdr*^R^ (1014F)-resistant *Anopheles gambiae*. KisKdr and Kisumu are, respectively, the homozygous *kdr*-resistant and -susceptible parents. F1-1 (♀ Kis X ♂ KisKdr) and F1-2 (♂ Kis X ♀ KisKdr) represent the first-generation heterozygous offspring. In panel A, *** and n.s. indicate, respectively, *p* = 2.2 × 10^–16^ and not significant. In panel B, *** indicate *p* = 8.10^–4^. The sample sizes were showed in brackets on the top of scatter dots

Overall, 84% (168/200) of KisKdr females and 34.75% (172/495) of Kisumu females subjected to the assays took a blood meal, as shown in Fig. 4A. The KisKdr females showed a significantly higher blood-feeding rate than the Kisumu ones (*χ*^*2*^ = 136.32, *df* = 1, *p* = 2.2 × 10^–16^). Interestingly, the offspring heterozygote [*kdr*^RS^] females F1-1 and F1-2 displayed also consistently higher per cent of blood-fed individuals (respectively, 74.74% (71/95) and 85.71% (90/105)) than that of Kisumu [*kdr*^SS^] individuals (*χ*^*2*^ = 121.89, *df* = 2, *p* = 2.2 × 10^–16^) (Fig. 4A). In all cases, mosquitoes harbouring the *kdr*^R^ allele at both homozygote and heterozygote states showed higher blood-feeding ability compared to the susceptible homozygote Kisumu strain (GLM: (RLT): *χ*^*2*^ = 215.28, *Δdf* = 2, *p* = 2.2 × 10^–16^).

When using other batches of mosquito females for the same blood-feeding assays, the average blood volume ingested by KisKdr individuals, was similar to that of Kisumu specimens (*p* = 0.22) while the average amount of blood ingested by the heterozygous offspring (1.68 µL/mg) was significantly higher than for Kisumu mosquitoes (1.36 µL/mg) (*p* = 8.10^–4^), as shown in Fig. 4B.

### Adult female survivorships post-blood feeding

**Fig. 5:**
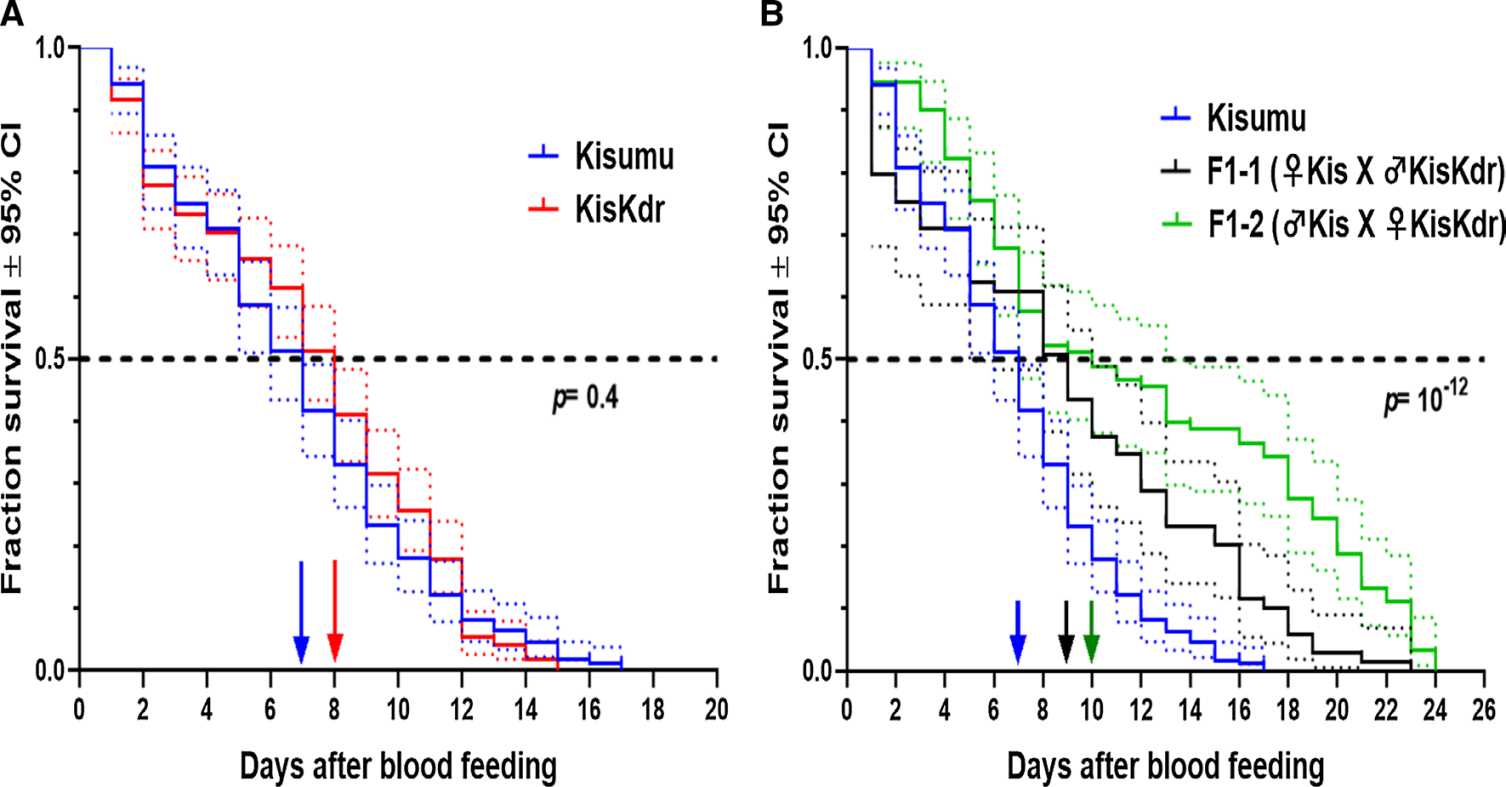
Parents and first generation female longevity after blood-feeding. Dotted lines are 95% confidence intervals (CIs) around the respective survival curve. Arrows indicate the median survival time

The median survival times after blood-feeding of the homozygous susceptible (Kisumu) and resistant (KisKdr) mosquitoes were, respectively, 7 days and 8 days (Fig. 5A). No significant difference in the survival time was observed between the two strains (Log-rank test: *χ*^*2*^ = 0.6, *Δdf* = 1, *p* = 0.4).

Moreover, the offspring heterozygote [*kdr*^RS^] displayed a longer median survival time after blood-feeding (10 days) compared to those of their parents (8 days for KisKdr; Log-rank test: *χ2* = 48, *Δdf* = 2, *p* = 4.10^–11^ and 7 days for Kisumu; Log-rank test: *χ2* = 54.9, *Δdf* = 2, *p* = 10^–12^). In addition, these offspring displayed a higher survival rate when compared to KisKdr females (hazard ratio = 0.44; Cox model: (LRT): *χ2* = 38.12, *Δdf* = 1, *p* = 7.10^–10^) and Kisumu specimens (hazard ratio = 0.41; Cox model: (LRT): *χ2* = 44.93, *Δdf* = 1, *p* = 2.10^–11^) as shown in Fig. 5A, B.

## Discussion

In the dominant malaria vector *An. gambiae*, pyrethroid resistance is spreading over time and space on the African continent, supported by several point mutations in the *Voltage-gated sodium channel* gene [21, 23, 51]. It was demonstrated that alleles conferring resistance in mosquito populations allow the mosquito to survive longer in an area of insecticide pressure but may alter some vector life-history traits [30, 31, 52] in an insecticide-free environment. Understanding and documenting the effects of *kdr* allele on life-history traits of *An. gambiae*, is a key for developing evidence-based resistance management strategies, including suppression of the insecticide selection pressure that allows the susceptible alleles to become more predominant [53].

This study has investigated the pleiotropic effects associated with the presence of the West African knockdown resistance allele (L1014F) on the reproductive success, larval and adult survivorships and blood-feeding success in laboratory *An. gambiae s.s.* by comparing the susceptible and resistant strains (homozygous *kdr* genotype) which share the same genetic background but differ in the presence or absence of the *kdr*^R^ (L1014F) allele. Reduced egg production and egg hatchability have been reported in other insecticide-resistant mosquito species, including *Ae. aegypti* [31, 54, 55]. However, in *An. funestus*, the egg production rates between pyrethroid-resistant and susceptible strains did not vary significantly [56]. The current study reported significantly lower fecundity and fertility in the homozygous KisKdr individuals compared to susceptible Kisumu strain mosquitoes. These results suggest that the *kdr*^R^ allele negatively affected the ability for egg production and hatchability in resistant homozygote [*kdr*^RR^] *An. gambiae*. Consequently, reduced larval production would reduce adult density and lead to a decreased level of malaria parasite transmission in the resistant *An. gambiae* mosquitoes.

This study revealed that the *kdr*^R^ (L1014F) allele confers a high larval-to-pupal survivorship and pupation rate in KisKdr mosquitoes compared to the susceptible strain. This suggests that both life-history traits are positively affected by the presence of the *kdr* allele. Relatively long larval development time and reduced survival time have previously been observed in insecticide-resistant *An. gambiae* [57]. It was recently demonstrated that insecticides in the larval environment (containing a lower dose of pyrethroid insecticide with a variation of food availability) could significantly influence the immune response of adults *An. gambiae* [58]. Indeed, the exposure of pyrethroid-resistant larvae (having escaped potential predators) to sub-lethal doses of insecticide residues during their aquatic developmental stage, especially in agricultural areas, could further affect the adult life-history traits. Such a phenomenon could drive the emergence of new outcomes related to the infection with specific mosquito-borne pathogens and the persistence of insecticide-resistant *An. gambiae*, which is still an essential impediment to the malaria vector control measures.

The results from this work indicate a significant association between harbouring of *kdr*^R^ allele and the high blood-feeding success in *An. gambiae s.s.*. This result suggests that the L1014F *kdr* allele may increase the ability of *An. gambiae* to blood-feed. By contrast, an absence of association was observed for the blood meal volume*.* Previous work on insecticide resistance markers has shown an association between the *CYP6P9a* gene (a marker of cytochrome P450, which mediates metabolic resistance again pyrethroid insecticides) and the feeding success and blood meal size in *An. funestus* [59]*.* These findings highlight the need for further studies to improve knowledge of the influence of multiple insecticide resistance markers harbouring on the propensity of malaria vectors to blood feed. However, heterozygous KisKdr F1-1 and F1-2 mosquitoes ingested higher blood volume compared to Kisumu specimens.

Gametocyte-infected mosquitoes must survive long enough to become infectious and transmit sporozoites to a new host [60]. One of the key factors modulating malaria transmission is the vector longevity after blood-feeding. This study demonstrates that the presence of *kdr*^R^ allele seems to increase the longevity of heterozygote KisKdr mosquitoes while no survival advantage was observed in homozygous individuals compared to the susceptible strain Kisumu. This benefit in heterozygote [*kdr*^RS^] over homozygote [*kdr*^RR^] makes the *kdr* an over-dominant gene for this specific trait. The heterozygote mosquitoes survived until 24 days post-blood meal. Thus, these specimens have sufficient lifespan to allow an extrinsic incubation period of *Plasmodium* parasites if they ingest gametocyte-infected blood. However, further investigations are needed to evaluate the cost of *Plasmodium* infection to heterozygote-resistant KisKdr mosquito survivorship.

## Conclusion

In order to generate valuable predictions of malaria transmission, the impact of resistance mechanisms on the vector life-history traits needs to be taken into consideration. The data presented here indicate that *kdr*^R^ allele induces a cost on fecundity and fertility in adult *An. gambiae*. Remarkably, this allele positively affects the larval survivorship, pupation rate, blood-feeding success in homozygote-resistant mosquitoes, and increases the post-blood feeding survivorship, especially in heterozygote individuals. It would be interesting to characterize the fitness effects of *kdr*^R^ allele in natural populations of *An. gambiae* and identify the potential synergist genes.

## Data Availability

The datasets are available from the corresponding author on reasonable request.

## Abbreviations

*Kdr*^*R*^: Resistant allele of knockdown resistance
*Kdr*^*S*^: Susceptible allele of knockdown resistance
*ace-1*^*R*^: Resistant allele of insecticide-insensitive acetylcholinesterase-1
*s.s*.: Sensu stricto
L1014F: Leucine substitution by phenylalanine at codon 1014
L1014S: Leucine substitution by serine at codon 1014
N1575Y: Asparagine-to-tyrosine substitution at codon 1575
LLINs: Long-lasting insecticide-treated nets
IRS: Indoor residual spraying
G119S: Glycine substitution by serine at codon 119
*Vgsc*: Voltage-gated sodium channel
GLM: Generalized linear models
NBM: Negative binomial model
MFAs: Membrane feeding assays
LRT: Likelihood ratio test
